# The Length Polymorphism of the 9th Intron in the Avian *CHD1* Gene Allows Sex Determination in Some Species of Palaeognathae

**DOI:** 10.3390/genes13030507

**Published:** 2022-03-12

**Authors:** Aleksandra Kroczak, Heliodor Wierzbicki, Adam Dawid Urantówka

**Affiliations:** Department of Genetics, Wrocław University of Environmental and Life Sciences, Kożuchowska 7, 51-631 Wrocław, Poland; aleksandra.kroczak@upwr.edu.pl (A.K.); heliodor.wierzbicki@upwr.edu.pl (H.W.)

**Keywords:** avian sex chromosomes, *CHD1* gene, molecular markers, Palaeognathae, sex typing

## Abstract

In palaeognathous birds, several PCR-based methods and a range of genes and unknown genomic regions have been studied for the determination of sex. Many of these methods have proven to be unreliable, complex, expensive, and time-consuming. Even the most widely used PCR markers for sex typing in birds, the selected introns of the highly conserved *CHD1* gene (primers P2/P8, 1237L/1272H, and 2550F/2718R), have rarely been effective in palaeognathous birds. In this study we used eight species of Palaeognathae to test three PCR markers: CHD1i9 (*CHD1* gene intron 9) and NIPBLi16 (*NIPBL* gene intron 16) that performed properly as Psittaciformes sex differentiation markers, but have not yet been tested in Palaeognathae, as well as the CHD1iA intron (*CHD1* gene intron 16), which so far has not been used effectively to sex palaeognathous birds. The results of our research indicate that the CHD1i9 marker effectively differentiates sex in four of the eight species we studied. In *Rhea americana*, *Eudromia elegans*, and *Tinamus solitarius*, the electrophoretic patterns of the amplicons obtained clearly indicate the sex of tested individuals, whereas in *Crypturellus tataupa*, sexing is possible based on poorly visible female specific bands. Additionally, we present and discuss the results of our in silico investigation on the applicability of CHD1i9 to sex other Palaeognathae that were not tested in this study.

## 1. Introduction

The infraclass Palaeognathae represented by the flightless ratites (ostrich, rhea, emu, cassowaries, kiwis, and the extinct New Zealand Moa) and the flying tinamous are considered a sister group of the Neognathae, which includes all other extant birds [1,2]. This division of modern birds (Neornithes), based on morphological and molecular data, has gained acceptance among evolutionary biologists [3,4,5]. Over 50 species of palaeognathous birds have been grouped into five orders (Apterygiformes, Casuariiformes, Struthioniformes, Rheiformes, and Tinamiformes) originating from various geographic landmasses (New Zealand, Australasia and Australia, Africa, and South America) [6]. Palaeognathous birds are characterized by a primitive skull morphology and the absence of a keel on the sternum, which makes them flightless (except for tinamous). A commonly accepted theory is that Palaeognathae originated in Gondwana, as the vast majority of existing species are found in the Southern Hemisphere [7,8]. However, another hypothesis has also emerged that Palaeognathae originated in the Northern Hemisphere after branching from Neognathae 104.7–115.5 million years ago [9]. They then extended their distribution by migrating to the Southern Hemisphere to lands originating in the former supercontinent Gondwana, such as New Zealand and Madagascar. Today, the flightless ratites inhabit Australia, Africa, and South America, whereas tinamous ratites are found only in South America [1].

Breeding programs of palaeognathous birds developed for commercial production of meat, leather, feathers, and oil (ostrich, emu, rhea), as well as in situ conservation programs and management of zoological collections require precise methods of sexing. This need is especially important for chicks and juveniles, which are usually monomorphic. According to Dawson et al. [10], in at least 50% of extant bird species, morphological differences do not allow for distinguishing their sex, and for most species, chicks cannot be sexed based on their morphology. Many of the developed methods of determining sex in birds are invasive (e.g., morphometric measurement, cloacal examination, laparotomy, and laparoscopy), which can be dangerous, especially for endangered species. Alternative methods, although not invasive (e.g., behavior observation, the ratio assay between estrogen and androgen in faces), tend to be time-consuming, often ambiguous, and imprecise [11,12,13]. Therefore, there was a need to develop an accurate method of sexing birds that would be noninvasive (or with a very limited invasiveness). Molecular tests based on DNA characteristics unique for each sex, especially those using PCR (Polymerase Chain Reaction) technology, have proven their reliability and simplicity. Additionally, they are inexpensive, fast, and have the advantage that DNA can be extracted from readily available samples such as feathers, buccal cells, fecal matter, and post-hatched egg-shell membrane [14,15,16].

In contrast to mammals with heterogametic males (XY) and homogametic females (XX), avian species are characterized by a sex chromosomal system with ZW heterogamety in females and ZZ homogamety in males. It is believed that regional suppression of recombination of a pair of autosomes led evolutionarily to the formation of the bird Z and W chromosomes [17,18]. Neognathous birds have significantly differentiated sex chromosomes with usually a smaller, degenerate, low gene content and highly heterochromatized W chromosome, whereas in palaeognathous birds, the Z and W chromosomes are often homomorphic or little differentiated with exceptionally large recombining pseudoautosomal regions [17,19,20]. The most basal forms of avian sex chromosomes recombine along most of their length occur in ratites, whereas in tinamous, signs of intermediate Z–W differentiation can be observed [21,22].

The female W chromosome should be an excellent source of sequences useful as genetic markers for sexing, as it is only found in females. Unfortunately, avian W chromosomes are usually degenerated, highly heterochromatic, and contain large amounts of repetitive sequences [23,24]. It is also known that rapidly evolving repetitive sequences are not a good source of sex-linked markers and, additionally, they work poorly in all types of DNA analyses [25]. However, it has been found that the sequences of more than 40 genes from the avian W chromosome contain a gametologous copy on the Z chromosome [26]. This allows sex determination because markers targeting W and Z copies of genes detect polymorphisms between them. When sexing birds with the use of PCR markers, in males a single product appears (genes copied from the two Z chromosomes are usually of identical length), whereas in females two products are visible due to the length polymorphism of the copies of genes located on the chromosomes Z and W [27]. To date, selected introns of the highly conserved *CHD1* (the Chromo Helicase DNA binding protein gene) have been the most studied and most often used as PCR markers for sexing avian taxa [28,29,30,31]. Other PCR markers also used for detecting the sex of birds have been developed based on the following genes: *NIPBL* (the Nipped-B homolog gene) [32], *SPIN* (the Spindlin gene) [33], and *RASA1* (the RAS p21 protein activator 1 gene) [34].

In palaeognathous birds, a number of PCR-based methods and a variety of genes and unknown genomic regions have been studied for sex determination (Table 1 and Supplementary Table S1). Many of these methods have proven to be unreliable, complex, expensive, and time-consuming. Additionally, no PCR-based method using only two primers has reliably determined the sex of palaeognathous birds (i.e., nonspecific amplicons produced, often not well seen, and not clearly separated bands distinguishing males—one band, and females—two bands; Supplementary Table S1). Even P2/P8, 1237L/1272H, and 2550F/2718R primers, which are the most widely used for sex typing in birds, in palaeognathous taxa were effective only in two cases (Table 1). However, to precisely determine the sex of the analyzed individuals, a polyacrylamide gel or more than two primers were required for effective testing.

In our previous study [14], carried out in Neognathae (135 species of the order Psittaciformes), we found that there is no single universal genetic marker useful for sexing a wide spectrum of avian species. We proposed a new sexing strategy based on multiple markers (four W/Z polymorphisms—CHD1i16, NIPBLi16, CHD1i9, and CHD1iE) that confirms the sex of a given individual with at least two markers detecting independent Z/W polymorphisms. The results of our research on sexing parrots [14] encouraged us to conduct research on the development of a simple PCR marker-based method for sexing palaeognathous birds. To do this, we selected two introns, CHD1i9 (*CHD1* gene intron 9) and NIPBLi16 (*NIPBL* gene intron 16) that performed properly as sex differentiating markers in the order Psittaciformes, but have not yet been tested in Palaeognathae. In addition, we also tested the CHD1iA intron (*CHD1* gene intron 16), which so far has not been used effectively to sex palaeognathous birds (Table 1, primers 2550F/2718R). Here, we report the results of this study.

## 2. Materials and Methods

### 2.1. Biological Samples and DNA Extraction

Thanks to the courtesy of Polish zoological gardens, blood and feather samples were collected from eight species of the Palaeognathae. The Wrocław Zoo provided us with blood samples from three individuals (one male, two females) of *Struthio camelus* (Struthioniformes), and feather samples from two individuals (one male, one female) of *Rhea pennata* (Rheiformes) and from two individuals (one male, one female) of *Casuarius casuarius* (Casuariiformes). Blood samples of three species of Tinamiformes: *Crypturellus tataupa* (two individuals—one male, one female)*, Eudromia elegans* (ten individuals—six males, four females), and *Tinamus solitarius* (four individuals—two males, two females), and feather samples of six individuals (one male, one female, four unknown sex) of *Dromaius novaehollandiae* (Casuariiformes) were collected from the Warsaw Zoo. The Chorzów Zoo provided us with blood samples from two individuals (one male, one female) of *Rhea americana* (Rheiformes).

Blood samples were taken as dry blood spots on a fiber filter for laboratory analysis to prevent the potential process of microbial breakdown of blood cells and genomic DNA degradation. They were then preserved in parafilm-sealed Eppendorf tubes at −20 °C until use to avoid dampness. In the case of freshly collected breast feather samples, the feather shafts were cut off and stored under the same conditions until use. Total DNA was extracted from both tissue types using the Sherlock AX Kit (A&A Biotechnology, Gdynia, Poland) according to the manufacturer’s protocol.

### 2.2. DNA Amplification

PCR amplifications were performed in 25 μL reaction mixture containing 50 ng of the DNA template, 1 U DreamTaq Green DNA Polymerase (Thermo Fisher Scientific, Waltham, MA, USA), 2.5 μL of 10 x buffer, 0.6 μL of 10 mM dNTPs, and 0.6 μL of each primer (10 μM).

We tested the use of CHD1iA, CHD1i9, and NIPBLi16 amplicons as potential markers for sexing palaeognathous taxa using the previously described PCR primers [32,40].

In most cases, the reaction conditions were as follows: **the CHD1iA program:** 94 °C 5 min, (94 °C 30 s, 50 °C 30 s, 72 °C 90 s) 35 times, and 72 °C 5 min; **the CHD1i9 program:** 94 °C 5 min, (94 °C 30 s, 51 °C 30 s, 72 °C 60 s) 35 times, and 72 °C 5 min; **the NIPBLi16 program:** 94 °C 5 min, (94 °C 30 s, 53 °C 30 s, 72 °C 60 s) 35 times, and 72 °C 5 min. For each amplified fragment, 5 ul of the PCR reaction mixtures was loaded onto the 1% agarose gel. In the case of *Crypturellus tataupa* and *Tinamus solitarius*, additional annealing temperatures (50 °C, 52 °C, 54 °C, 56 °C, and 56 °C, respectively) were tested for the CHD1i9 amplicon.

## 3. Results and Discussion

Sex-determining PCR markers developed for neognathous birds have often failed to determine the sex of species belonging to their sister taxon, the Palaeognathae. This is due to the evolutionary divergence and ancestral state of the sex chromosomes in Palaeognathae birds [41]. Therefore, the search for PCR markers and PCR-based methods, the application of which for sex-typing in the Palaeognathae would be simple, highly sensitive, fast, and cost-effective, is of constant interest to people involved in the creation of breeding and conservation programs as well as scientific research of these birds (especially those that are endangered). A review of standard PCR-based methods used for sex determination of ratites shows that most of them are species-specific, and a significant number of W-linked markers have been developed using the RAPD method [41] (Supplementary Table S1). Research on the development of a single universal sex differentiating marker in ratites has shown some limitations in the accuracy and reliability of sexing with selected primers [42,43].

With this in mind, we tested three W/Z polymorphisms (CHD1i9, NIPBLi16, and CHD1iA), the first two of which have never been studied before in palaeognathous birds. When assessing their usefulness, we focused the most on their reliability (sensitivity) in determining the sex of the bird. Other factors important when sexing birds, such as time and cost efficiency and simplicity of the method, were retained because the standard PCR method was used. The positions of CHD1i9, CHD1iA, and NIPBLi16 within the *CHD1* and *NIPBL* genes in *Gallus gallus* sex chromosomes were illustrated in our previous study [14].

### 3.1. CHD1iA and NIPBLi16 Markers Are Not Suitable for Sex Determination of Palaeognathous Birds

**Figure 1:**
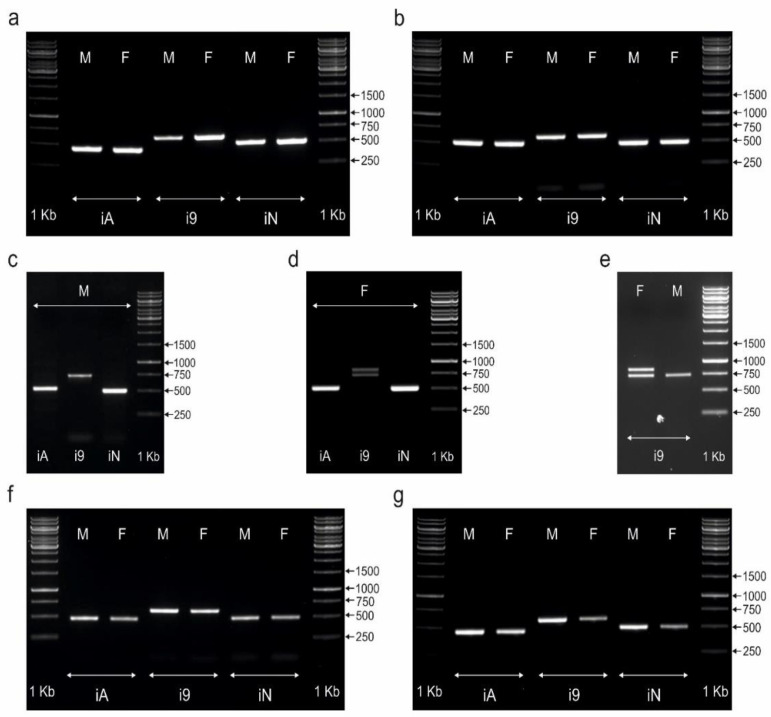
PCR products obtained for male (M) and female (F) individuals of *Struthio camelus* (**a**), *Rhea pennata* (**b**), *Rhea americana* (**c**,**d**,**e**), *Dromaius novaehollandiae* (**f**), and *Casuarius casuarius* (**g**) species using markers CHD1iA (iA), CHD1i9 (i9), and NIPBLi16 (iN). The amplicons were resolved in 1% agarose gel. Arrows and numbers correspond to the location and size (in bp) of the DNA molecular marker (1 kb) run on the same gel.

**Figure 2:**
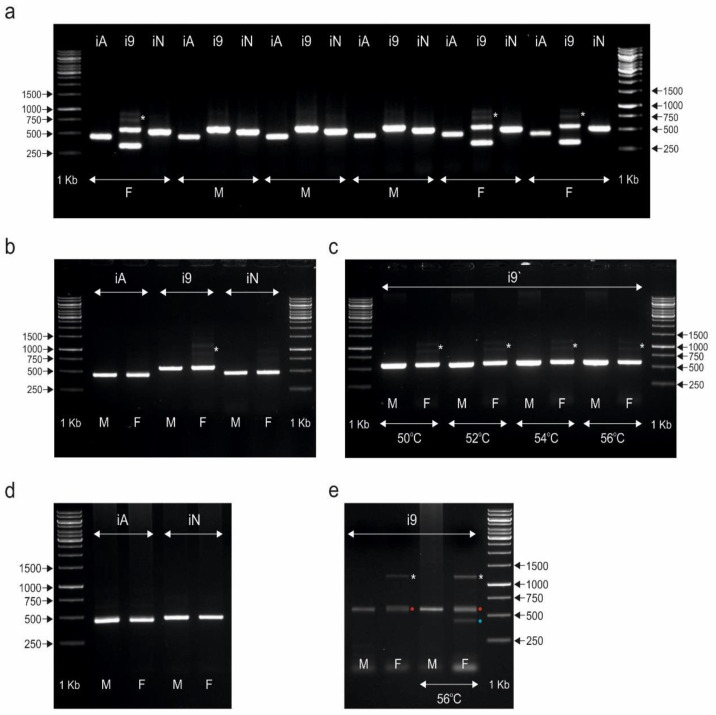
PCR products obtained for male (M) and female (F) individuals of *Eudromia elegans* (**a**), *Crypturellus tataupa* (**b**,**c**), and *Tinamus solitarius* (**d**,**e**) species using CHD1iA (iA), CHD1i9 (i9), and NIPBLi16 (iN) markers. The amplicons were resolved in 1% agarose gel. Arrows and numbers correspond to the location and size (in bp) of the DNA molecular marker (1 kb) run on the same gel. In the case of the CHD1i9 marker, annealing temperatures other than 51 °C are marked appropriately if they were used. Asterisks, red and blue dots indicate additional bands detected only in females.

In our study of eight species of palaeognathous birds, the CHD1iA and NIPBLi16 markers did not distinguish between males and females (Figure 1 and Figure 2). Sex determination with both tested markers was not possible, as identical patterns were obtained after PCR in both sexes (only one band was amplified). For the CHD1iA marker, only one PCR product was detected, ranging in length from 480 bp (*Struthio camelus*, *Dromaius novaehollandiae*, *Casuarius casuarius*, *Crypturellus tataupa*, and *Tinamus solitarius*), followed by 490 bp (*Eudromia elegans*) to 500 bp (*Rhea pennata*, *Rhea americana).* The second band was not seen most likely due to the lack of difference between the lengths of the A introns located in the Z and W copies of the *CHD1* gene. It was also possible that their length polymorphism was so weak that it was impossible to distinguish between the two copies of intron A on the 1% agarose gel. A similar situation was observed in the case of the NIPBL1i16 marker, which also turned out to be unsuitable for sexing the studied bird species. Only one band of this marker with a length ranging from 490 bp (*Dromaius novaehollandiae* and *Crypturellus tataupa*) to 500 bp (*Struthio camelus*, *Rhea pennata*, *Rhea americana*, *Casuarius casuarius*, *Eudromia elegans*, and *Tinamus solitarius*) was detected and visualized. The patterns produced by the markers CHD1iA and NIPBL1i16 for *Struthio camelus*, *Rhea pennata*, *Rhea americana*, *Dromaius novaehollandiae*, and *Casuarius casuarius* are shown in Figure 1, whereas those produced for *Eudromia elegans*, *Crypturellus tataupa*, and *Tinamus solitarius* are shown in Figure 2.

So far, sexing of selected palaeognathous birds (*Dromaius novaehollandiae, Casuarius casuarius, Rhea americana*) with the use of the CHD1iA marker (PCR primers 2550F/2718R), as in our study, has been unsuccessful [29,37] (see Table 1). According to Fridolfsson and Ellegren [40], the difference between the Z and W bands resulting from the amplification with 2550F/2718R primers should be in the range from 150 to 250 bp, allowing high-quality separation and visualization on a 1% agarose gel. However, in the studies cited above, a single band of the same size was observed in all samples of the sexed species. The lack of heteromorphic sex chromosomes seems to be the most likely cause of failure in the molecular sexing of these species [29].

Regarding the use of the NIPBL1i16 marker for sexing palaeognathous birds, to our knowledge, it has not yet been used for this purpose. The application of NIPBLi16 as a sexing marker in birds is based on the presence or absence of the CR1 (chicken repeat 1 family of long interspersed elements) retroposon insertion in the Z or W chromosomes, respectively [32]. According to Suh et al. [32], given the significant differences in the length of the Z and W amplicons (400 bp in intron 16 of the *NIPBL* gene), this marker should allow for a convenient molecular determination of Neoaves gender. However, in palaeognathous birds, the Z and W amplicons show no distinct size differences. Indeed, in our previous study [14] conducted on 135 species of parrots (Psittaciformes), of the six gender differentiation markers tested (P2P8, CHD1iA, CHD1iE, CHD1i16, CHD1i9, and NIPBLi16), the NIPBLi16 marker was the most effective, allowing 128 of the 135 species tested to be sexed. However, the results of the present study on the use of this marker for sexing eight species of Palaeognathae showed that it is not suitable for this group of birds. The reason for this, as suggested by Suh et al. [32], may be the lack of the insertion of Z gametologous retroposon in earlier branching species (e.g., those belonging to Palaeognathae).

### 3.2. The CHD1i9 Marker Enables Sex Determination in Selected Species of Palaeognathous Birds

When investigating retroposon insertions and avian sex chromosome evolution, Suh et al. [32] recommended the CHD1i9 marker (and two other markers: CHD1i16 and NIPBLi16) for the molecular determination of sex of Neognathae species. This was due to the detection of a larger product of PCR amplification of the CHD1 intron 9 in the Z chromosome as compared to the W chromosome (Z and W amplicon size difference = 500 bp). This was explained by the insertion of the LTR retroposon (long terminal repeat elements of endogenous retroviruses) in the CHD1i9 copy located on the Z chromosome. However, the authors did not present these results or refer to the potential use of this marker in determining the sex of palaeognathous birds. As this marker has not yet been tested for sex determination in Palaeognathae, we decided to test it on eight species belonging to this infraclass.

The results of our study are presented in Figure 1 and Figure 2. In four out of eight tested species, sex determination using the CHD1i9 marker was not possible, as identical banding patterns were obtained in both female and male samples. The species for which only one amplicon (600–650 bp) was detected were: *Struthio camelus*, *Rhea pennata*, *Dromaius novaehollandiae*, and *Casuarius casuarius* (Figure 1). In the remaining four species (*Rhea americana*, *Eudromia elegans*, *Crypturellus tataupa*, and *Tinamus solitarius*) we obtained interesting results suggesting that CHD1i9 may be useful for sexing other Palaeognathae taxa.

Using the CHD1i9 marker, sex could be distinguished in *Rhea americana* (Figure 1c,d,e), although the same marker did not allow sexing of a species belonging to the same genus—*Rhea pennata*. Males of *Rhea americana* produced one 750 bp Z-specific amplicon, whereas females produced both CHD1Z (750 bp) and CHD1W (~850 bp) amplicons. The PCR products that appear in females are clearly visible and separated in 1% agarose gel, allowing for reliable sex determination. Compared to *Rhea pennata*, the amplicon obtained for the male *Rhea americana* is approximately 100–150 bp larger (600–650 bp vs. 750 bp, respectively).

PCR amplifications of CHD1i9 in *Eudromia elegans* (Figure 2a) showed two distinct amplicons in females (Z-specific band ~ 580 bp, and W-specific band ~ 300 bp) and only one Z-specific band (~580 bp) in males. Additionally, two less visible bands were detected only in females (marked with an asterisk)—one visible slightly below 750 bp of the DNA ladder and the other visible slightly above 750 bp of the DNA ladder. One might speculate why in the female banding pattern additional faintly visible bands were detected. If we assume that the W-chromosome located CHD1i9 with the inserted LTR retroposon was duplicated and/or relocated and then some mutations occurred in the primer binding sites of the copied sequence, this could have altered the primer annealing ability. In this case, additional PCR products (of different lengths) may have appeared as faintly visible bands. However, further research is needed to elucidate and confirm this.

In the case of *Crypturellus tataupa*, only one clearly visible band of ~580 bp was detected for both sexes. Interestingly, as in the case of *Eudromia elegans*, two additional shadow bands (marked with an asterisk) were visible only in females (Figure 2b). One of the faintly visible extra bands in females is visible just above and the other is visible just below 1000 bp of the DNA ladder. However, the lengths of these additional bands detected in *Crypturellus tataupa* are different from those seen in *Eudromia elegans* (~750 bp). The use of several non-standard (51 °C) annealing temperatures (50 °C, 52 °C, 54 °C, and 56 °C) did not change the pattern characterizing the female *Crypturellus tataupa* (Figure 2c).

The most complex pattern of amplicons was found when testing CHD1i9 as a gender differentiation marker in *Tinamus solitarius* (Figure 2e). The Z-specific band with a length ~580 bp is clearly visible in males and females. However, at the standard annealing temperature (51 °C), only in the female sample was an additional (probably W-specific), slightly longer amplicon with a length of ~600 bp obtained. This additional amplicon is hardly visible on the 1% agarose gel (marked with a red dot—Figure 2e) due to the slight difference in length between the two fragments. Moreover, a clearly visible band above 1000 bp of the DNA ladder also appears only in females (marked with an asterisk, Figure 2e). Its length correlates with the molecular mass of the larger shadow band obtained for *Crypturellus tataupa* (Figure 2b,c). At a higher annealing temperature (56 °C), the pattern with a Z-specific band (~580 bp) is maintained for males. However, an additional band (marked with a blue dot) with a length of ~ 420 bp was amplified for the female sample. In summary, the presence of a female-specific fragment of approximately 1200 bp indicates that CHD1i9 can be effectively used in the sexing of this species, especially as its amplification is independent of the annealing temperature tested (in contrast to the 420 bp amplicon which is only observed at the annealing temperature of 56 °C, Figure 2e).

In our study, we tested only three species (*Crypturellus tataupa, Eudromia elegans, Tinamus solitaries)* of 46 belonging to the order Tinamiformes. Therefore, it is possible that CHD1i9 would be an effective sexing marker for many other tinamous that we have not tested. To verify this, we performed the following in silico investigation.

First, to be able to search the available sequences of nuclear genomes, we designed an appropriate reference sequence. As a starting point, we used the *CHD1* gene sequence (accession number: NW_004973325.1) found with the gene database in the nuclear genome available for *Columba livia* (a species that is not closely related to the Palaeognathae), and then downloaded it in the FASTA format. The length of the downloaded sequence was 60,753 bp. In this sequence containing the *CHD1* gene, using a sequence alignment, we determined the positions of primers designed to amplify the ninth intron of the *CHD1* gene (primers described by Suh et al. [32]).

Second, using the designed reference sequence (it was our query), we searched the nuclear genomes of selected palaeognathous birds available in the database. We used the Basic Local Alignment Search Tool (BLAST) for searching genomes. We found the sought sequence in the genomes of three representatives of the genus Apteryx (*Apteryx rowi, Apteryx haastii*, and *Apteryx owenii)* and in eight tinamous of the genera: Crypturellus, Eudromia, Nothocercus, Nothoprocta, and Guttatus (Table 2). Most genomes were obtained for males, and in most of the taxa only one sequence, approximately 600 bp long, was found. However, in three species, *Crypturellus soui*, *Crypturellus undulates*, and *Tinamus guttatus*, two copies of the ninth intron in the *CHD1* gene were identified. In these species, the sequences were from females, so we suspect that each of the copies found comes from a different sex chromosome: one from the W chromosome (probably this shorter) and the other (similar in size to the sequence found in males) from the Z chromosome. However, we are uncertain which of the two copies is Z-specific and which is W-specific. Our findings are summarized in Table 2.

Third, using the MUSCLE algorithm [44] of the MEGA program [45], the sequences of both copies of the ninth intron of the *CHD1* gene found for *Crypturellus soui*, *Crypturellus undulatus*, and *Tinamus guttatus* were compared. The results of this alignment are visualized in Supplementary Figure S1. The copies of the ninth intron in the *CHD1* gene identified in *Crypturellus soui* differ significantly in length. However, one of the copies (230 bp) is an incomplete sequence, and we do not know its true length. Nevertheless, the alignment of the ninth intron obtained for this species showed that the two copies had different sequences. In addition to the altered nucleotides, it can be seen that there were deletions within them. In *Crypturellus undulates*, the copies of the ninth intron of the *CHD1* gene are very similar in length (601 bp vs. 609 bp); however, when analyzing their alignment, changes in the sequences of both copies can be noticed. Traces of deletion are also noticeable. In the case of *Tinamus guttatus*, both sequences differ in length (the difference between 603 and 583 bp would probably be seen in an agarose gel). The two copies also have different sequences. Changes in nucleotide composition and a deletion in the shorter copy are visible. These results indicate that it would likely be possible to use the length polymorphism of the ninth intron in the *CHD1* gene to determine the sex of other tinamous that we have not studied (laboratory tests are needed to verify this).

In total we studied eight species of Tinamiformes—three of them (*Crypturellus tataupa, Tinamus solitarius*, and *Eudromia elegans*) were tested in the laboratory, and an additional five species (*Crypturellus soui*, *Crypturellus undulatus*, *Nothocercus nigrocapillus, Nothoprocta pentlandii*, and *Tinamus guttatus*) were investigated in silico (in the database, we found sequences for eight tinamous species, but only five of them were females and their sequences were analyzed). Analysis of several sequences available in the database for females indicates that differences in CHD1i9 length may also exist. Although the introns we found in the database differ in sequence rather than length, it should not be ruled out that in the remaining tinamous species that we have not studied, the differences in length are more pronounced.

In summary, we tested only 8 out of 46 species of Tinamiformes, so in the remaining 38 species it would be necessary to test whether the length polymorphism of the ninth intron of the *CHD1* gene is comparable to that observed in *Eudromia elegans*. If that were the case, it would enable visualization of the obtained amplicons in a 1% agarose gel and effective sexing.

In our opinion, it is also worth investigating whether the length polymorphism of the ninth intron in the *CHD1* gene allows for determining the sex of five species currently recognized in the order Apterygiformes. This cannot be ruled out, as the p2 and p8 primers of the *CHD1* gene allow for determining the sex of *Apteryx mantelli* (Table 1 [35]). Unfortunately, we did not have such a possibility in this study. Furthermore, it is not possible to verify in silico if two different copies of CHD1i9 are present on the sex chromosomes of any *Apteryx* species, as so far the nuclear genomes representative of the taxa *Apteryx rowi*, *Apteryx haastii*, and *Apteryx owenii* have only been sequenced for males (GenBank Biosamples: SAMN08476454, SAMN08476452, and SAMN08476453).

## 4. Conclusions

The number of PCR markers used for sex determination of Palaeognathae is very limited due to the evolutionary divergence and ancestral state of their sex chromosomes. Therefore, new PCR markers that allow rapid and accurate sexing of these birds are highly anticipated. The results of our research indicate that the CHD1i9 marker, which has not yet been used to sex Palaeognathae birds, effectively differentiates sex in four of the eight species we studied. In *Rhea americana*, *Eudromia elegans*, and *Tinamus solitarius*, the electrophoretic patterns of amplified fragments clearly indicate the sex of the individuals tested, whereas in *Crypturellus tataupa*, sexing is only possible based on additional, faintly visible female-specific bands.

The effective use of the CHD1i9 marker for sexing the four Palaeognathae species we studied seems to be a promising prospect for the use of this marker in sex determination of other species of the order Tinamiformes. In addition, we believe that the length polymorphism of the ninth intron in the *CHD1* gene may allow sexing individuals belonging to the order Apterygiformes. However, this requires further research.

## Figures and Tables

**Table 1:** Molecular markers based on the intron length polymorphism of the *CHD1* gene used for sex determination in Palaeognathae species.

Order	Species	Primers	Amplicons M/F	Impracticality	Utility	References
Apterygiformes	*Apteryx mantelli*	P2/P8	1/2	separation in 6% polyacrylamide	+	[35]
Casuariiformes	*Dromaius novaehollandiae*	1237L/1272H	1/1	any female specific band	−	[29,36]
Casuariiformes	*Dromaius novaehollandiae*	2550F/2718R	1/1	any female specific band	−	[29,37]
Casuariiformes	*Casuarius casuarius*	2550F/2718R	1/1	any female specific band	−	[37]
Rheiformes	*Rhea americana*	2550F/2718R	1/1	any female specific band	−	[37]
Struthioniformes	*Struthio camelus*	P2/P8 + P0	1/2	3 primers are used	+ +	[38]
Struthioniformes	*Struthio camelus*	1237L/1272H	1/1	any female specific band	−	[29,36]
Struthioniformes	*Struthio camelus*	2550F/2718R	1/1	any female specific band	−	[29,30]
Struthioniformes	*Struthio camelus*	P2/P8	1/1	any female specific band	−	[39]

**Table 2:** Kiwi and Tinamous species analysed in this study in terms of length polymorphism of the ninth intron in the *CHD1* gene. The accession numbers and length of the identified fragments are shown, as well as the assigned sex and biosample numbers. A single (*) or double (**) asterisk indicates different copies of the ninth intron in the *CHD1* gene identified for *Crypturellus soui*, *Crypturellus undulates*, and *Tinamus guttatus*. A copy with only a partial sequence identified for *Crypturellus toui* is underlined.

Species	Sex	Biosample	CHD1i9	
			Accession	Length (bp)
*Apteryx rowi*	Male	SAMN08476454	NW_020450197.1	632
*Apteryx haastii*	Male	SAMN08476452	PTFD01000391.1	627
*Apteryx owenii*	Male	SAMN08476453	PTFC01000295.1	632
*Crypturellus cinnamomeus*	Male	SAMN08476456	PTEZ01000258.1	601
*Crypturellus soui*	Female	SAMN12253745	VWPX01020692.1	598 *
*Crypturellus soui*	Female	SAMN12253746	VWPX01026815.1	220 **
*Crypturellus undulatus*	Female	SAMN12253747	VWPW01009891.1	601 *
*Crypturellus undulatus*	Female	SAMN12253746	VWPW01026715.1	609 **
*Eudromia elegans*	Male	SAMN08476458	PTEX01000066.1	579
*Nothocercus nigrocapillus*	Female	SAMN12253973	WBNA01000102.1	590
*Nothoprocta pentlandii*	Female	SAMN12253975	VZSG01000618.1	596
*Nothoprocta perdicaria*	Male	SAMN08476459	NW_020455588.1	598
*Tinamus guttatus*	Female	SAMN02316659	NW_010585320.1	603 *
*Tinamus guttatus*	Female	SAMN02316660	NW_010577858.1	583 **

## Data Availability

Our research does not involve any nucleotide or amino acid sequence data that should be archived in publicly accessible repositories. All PCR patterns obtained are presented in figures included in the manuscript.

## Supplementary Materials

The following supporting information can be downloaded at: https://www.mdpi.com/article/10.3390/genes13030507/s1, Table S1: Methods used for the molecular sexing of Palaeognathae species; Figure S1: Sequence comparison of two copies of the ninth intron in the *CHD1* genes found in this study. The alignments are shown for *Crypturellus soui* (A), *Crypturellus undulatus* (B), and *Tinamus guttatus* (C). Dots indicate residues identical in both copies. The sequences were aligned with MUSCLE [44] in MEGA [45]. References [46,47,48,49,50,51,52,53,54,55,56,57,58,59,60,61] are cited in the supplementary materials.
