# Supplementary material for: The Length Polymorphism of the 9th Intron in the Avian CHD1 Gene Allows Sex Determination in Some Species of Palaeognathae
Source: Genes (Basel). 2022 Mar 12;13(3):507. doi: 10.3390/genes13030507 (PMC8954394; doi:10.3390/genes13030507)
Supplement: Supplementary file 1 [file genes-13-00507-s001.zip › Supplementary-files/Supplementary_Figure S1.pdf]

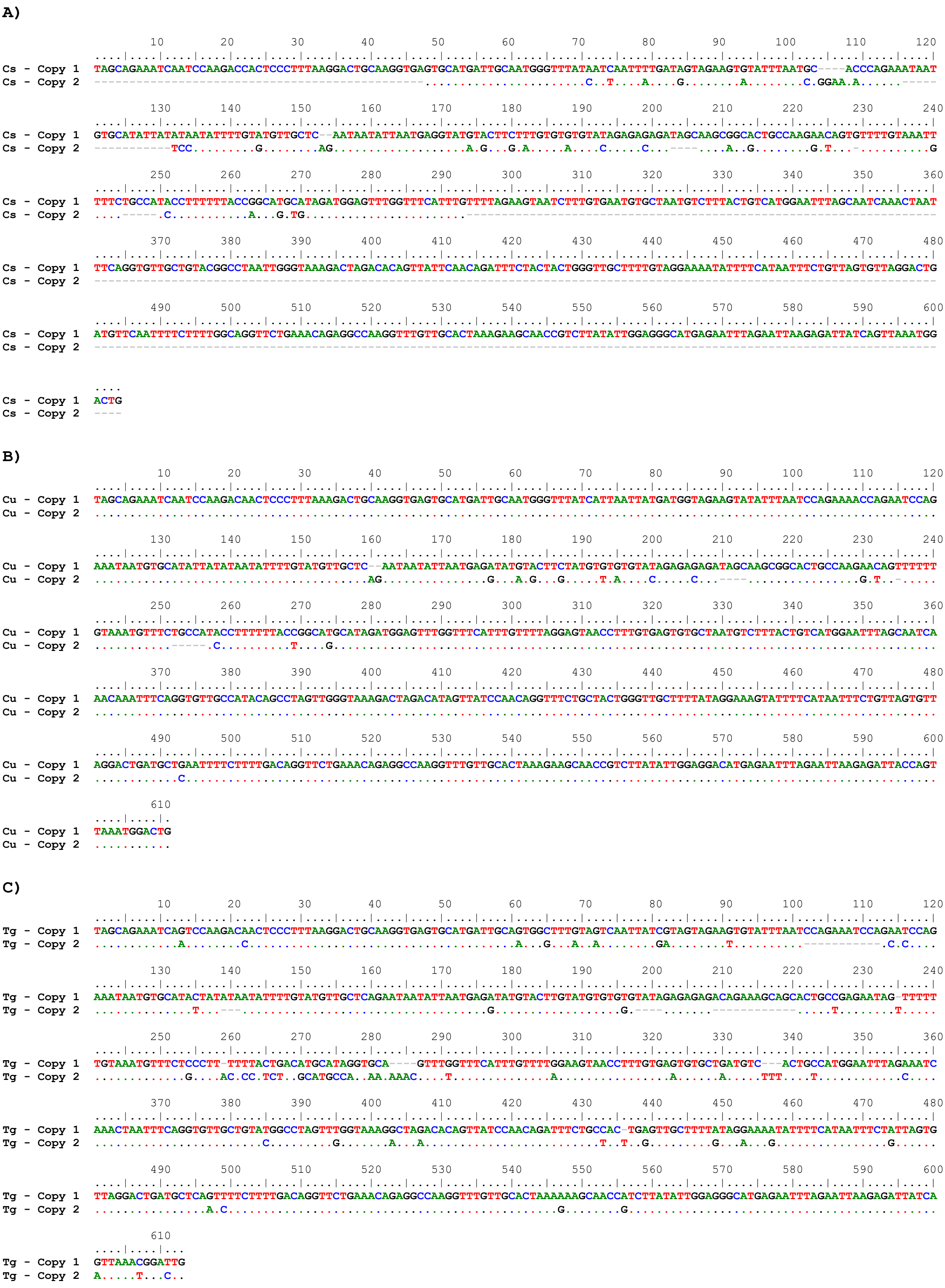

Figure S1. Sequence comparison of two copies of the 9th intron in the CHD1 gene found in this study. The alignments are shown for *Crypturellus soui* (A), *Crypturellus undulatus* (B), and *Tinamus guttatus* (C). Dots indicate residues identical in both copies. The sequences were aligned with MUSCLE [44] in MEGA [45].
